# Dimethyl 5-amino-2,4,6-triiodo­isophthalate

**DOI:** 10.1107/S1600536810001005

**Published:** 2010-01-16

**Authors:** Pei Zou, Shi-Neng Luo, Min-Hao Xie, Ya-Ling Liu, Jun Wu

**Affiliations:** a Jiangsu Institute of Nuclear Medicine, Wuxi 214063, People’s Republic of China

## Abstract

The title compound, C_10_H_8_I_3_NO_4_, crystallizes with two mol­ecules in the asymmetric unit. The I atoms and the benzene ring plane in the two mol­ecules are approximately coplanar, the I atoms deviating by −0.1631 (1), 0.0704 (1) and −0.0507 (1) Å from the mean plane of the benzene ring in one mol­ecule and by 0.1500 (1), −0.0034 (1) and −0.1213 (1) Å in the other. The planes of the ester groups are almost orthogonal to those of the benzene rings in both mol­ecules, forming dihedral angles of 83.5 (3), 76.4 (3), 97.3 (1) and 75.7 (1)°. The mean planes of the benzene rings in two mol­ecules are inclined at 69.8 (3)° with respect to each other. In the crystal, inter­molecular I⋯O inter­actions link the mol­ecules into infinite chains. In addition, N—H⋯O and non-classical C—H⋯O hydrogen bonds are observed.

## Related literature

For general background to 1,3,5-triiodobenzene derivatives, see: Morin *et al.* (1987 ▶); Singh & Rathore (1980 ▶); Stacul *et al.* (2001 ▶); Yu & Watson (1999 ▶). For a related structure, see: Beck & Sheldrick (2008 ▶).
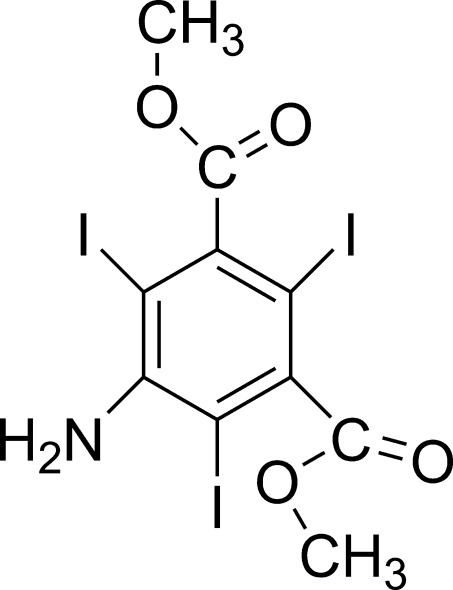


## Experimental

### 

#### Crystal data

C_10_H_8_I_3_NO_4_
*M* *_r_* = 586.87
Triclinic, 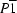

*a* = 8.4423 (17) Å
*b* = 10.3545 (19) Å
*c* = 18.365 (3) Å
α = 75.158 (5)°
β = 80.045 (5)°
γ = 89.728 (6)°
*V* = 1527.2 (5) Å^3^
*Z* = 4
Mo *K*α radiation
μ = 6.15 mm^−1^
*T* = 93 K
0.40 × 0.33 × 0.13 mm

#### Data collection

Rigaku SPIDER diffractometer
Absorption correction: empirical (using intensity measurements) (North *et al.*, 1968 ▶) *T*_min_ = 0.193, *T*_max_ = 0.495
10344 measured reflections
5251 independent reflections
4488 reflections with *I* > 2σ(*I*)
*R*_int_ = 0.036

#### Refinement

*R*[*F* ^2^ > 2σ(*F* ^2^)] = 0.035
*wR*(*F* ^2^) = 0.067
*S* = 0.98
5251 reflections
325 parameters
24 restraints
H-atom parameters constrained
Δρ_max_ = 1.10 e Å^−3^
Δρ_min_ = −1.19 e Å^−3^

### 

Data collection: *RAPID-AUTO* (Rigaku, 2004 ▶); cell refinement: *RAPID-AUTO*; data reduction: *RAPID-AUTO*; program(s) used to solve structure: *SHELXS97* (Sheldrick, 2008 ▶); program(s) used to refine structure: *SHELXL97* (Sheldrick, 2008 ▶); molecular graphics: *SHELXTL* (Sheldrick, 2008 ▶); software used to prepare material for publication: *SHELXTL*.

## Supplementary Material

Crystal structure: contains datablocks I, global. DOI: 10.1107/S1600536810001005/pv2251sup1.cif

Structure factors: contains datablocks I. DOI: 10.1107/S1600536810001005/pv2251Isup2.hkl

Additional supplementary materials:  crystallographic information; 3D view; checkCIF report

## Figures and Tables

**Table 1:** Hydrogen-bond geometry (Å, °)

*D*—H⋯*A*	*D*—H	H⋯*A*	*D*⋯*A*	*D*—H⋯*A*
N1*A*—H1*A*⋯I2*A*	0.88	2.74	3.224 (5)	116
N1*A*—H1*A*⋯O4*B*^i^	0.88	2.48	3.036 (7)	122
N1*A*—H1*B*⋯I3*A*	0.88	2.72	3.211 (5)	117
N1*B*—H1*C*⋯I2*B*	0.88	2.73	3.212 (5)	116
N1*B*—H1*D*⋯I3*B*	0.88	2.73	3.222 (5)	116
N1*B*—H1*D*⋯O2*A*^ii^	0.88	2.43	3.026 (7)	125
C8*B*—H8*E*⋯O2*B*^iii^	0.98	2.54	3.516 (9)	171
C10*A*—H10*A*⋯O2*B*^iv^	0.98	2.58	3.499 (9)	155
C10*A*—H10*B*⋯O4*A*^v^	0.98	2.54	3.519 (9)	173

Symmetry codes: (i) 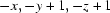; (ii) 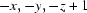; (iii) 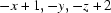; (iv) 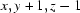; (v) 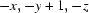.

**Fig. 1.**
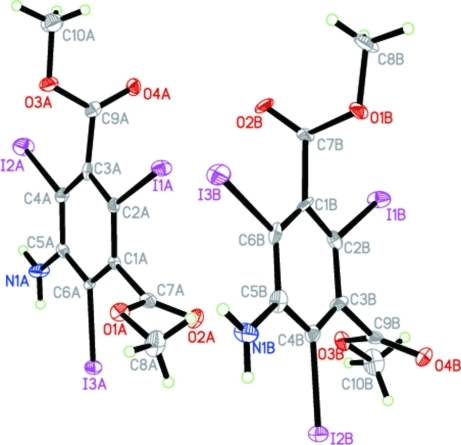
A view of the title compound with the atomic numbering scheme. Displacement ellipsoids were drawn at the 50% probability level.

**Fig. 2.**
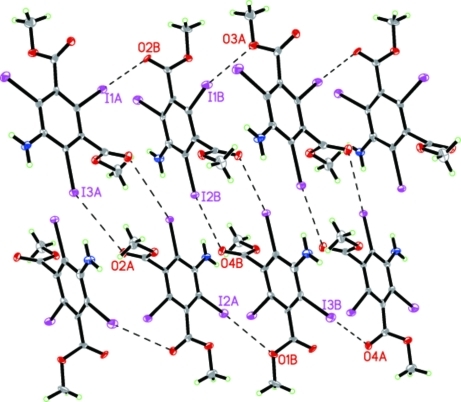
Partial view of molecular structure. Molecules are linked into infinite one dimensional chains by I···O interactions (dashed lines).

## supplementary crystallographic information

### Comment

The 1,3,5-triiodobenzene core has been the basis of many contrast agents (Yu & Watson, 1999). The title compound is useful as an important intermediate for the preparation of iodinated *X*-ray contrast agent, such as iotalamic acid, ioxitalamic acid, and ioxilan, which are used clinically all over the world (Morin *et al.*, 1987; Singh *et al.*, 1980; Stacul *et al.*, 2001). In this paper, we present the crystal structure of the title compound.

The asymmetric unit of the title compound (Fig. 1) contains two crystallographically independent molecules (A and B) in an asymmetric unit. The three I atoms deviate from the mean-planes of the phenyl rings, respectively, by -0.1631 (1), 0.0704 (1) and -0.0507 (1) Å for molecule A and 0.1500 (1), -0.0034 (1) and -0.1213 (1) Å for molecule B. Bond lengths and angles are comparable to those observed in a related structure (Beck & Sheldrick, 2008). The planes of the ester groups in both molecule are almost orthogonal to the benzene ring, as indicated by the dihedral angles of 83.5 (3)° (C10A/O3A/C9A/O4A; C1A—C6A), 76.4 (3)°(C8A/O1A/C7A/O2A; C1A—C6A), 97.3 (1)° (C10B/O3B/C9B/O4B; C1B—C6B) and 75.7 (1)° (C8B/O1B/C7B/O2B; C1B—C6B). The dihedral angle between the rings (C1A—C6A) and (C1B—C6B) is 69.8 (3)°.

In the crystal structure, intermolecular I···O interactions link the molecules into infinite one-dimensional chains (Fig. 2). In addition, C—H···O hydrogen bonds and N—H···O hydrogen bonds are observed.

### Experimental

A mixture of 5-amino-2,4,6-triiodoisophthaloyl dichloride (2.97 g, 5 mmol) and methanol (15 ml) was heated under reflux for four hours to produce dimethyl 5-amino-2,4,6-triiodoisophthalate. It was recrystallized from a methanol solution by slowly evaporating the solvents to obtain crystals suitable for X-ray single-crystal diffraction.

### Refinement

All H atoms were initially located from a difference Fourier map and then were regenerated at ideal positions and treated as riding, with N—H = 0.88 Å, C—H = 0.98 Å and *U*_iso_(H) = 1.2*U*_eq_ (N), *U*_iso_(H) = 1.5*U*_eq_ (C). The final difference map showed electron density in the vicinity of I3B atom and was deemed meaningless.

### Crystal data

**Table:**

C_10_H_8_I_3_NO_4_	*Z* = 4
*M**_r_* = 586.87	*F*(000) = 1064
Triclinic, *P*1	*D*_x_ = 2.553 Mg m^−^^3^
Hall symbol: -P 1	Mo *K*α radiation, λ = 0.71073 Å
*a* = 8.4423 (17) Å	Cell parameters from 4796 reflections
*b* = 10.3545 (19) Å	θ = 3.1–27.5°
*c* = 18.365 (3) Å	µ = 6.15 mm^−^^1^
α = 75.158 (5)°	*T* = 93 K
β = 80.045 (5)°	Chunk, colorless
γ = 89.728 (6)°	0.40 × 0.33 × 0.13 mm
*V* = 1527.2 (5) Å^3^	

### Data collection

**Table:**

Rigaku SPIDER diffractometer	5251 independent reflections
Radiation source: Rotating anode	4488 reflections with *I* > 2σ(*I*)
graphite	*R*_int_ = 0.036
ω scans	θ_max_ = 25.0°, θ_min_ = 3.1°
Absorption correction: empirical (using intensity measurements) (North *et al.*, 1968)	*h* = −7→10
*T*_min_ = 0.193, *T*_max_ = 0.495	*k* = −12→12
10344 measured reflections	*l* = −21→21

### Refinement

**Table:**

Refinement on *F*^2^	Primary atom site location: structure-invariant direct methods
Least-squares matrix: full	Secondary atom site location: difference Fourier map
*R*[*F*^2^ > 2σ(*F*^2^)] = 0.035	Hydrogen site location: inferred from neighbouring sites
*wR*(*F*^2^) = 0.067	H-atom parameters constrained
*S* = 0.98	*w* = 1/[σ^2^(*F*_o_^2^) + (0.0226*P*)^2^] where *P* = (*F*_o_^2^ + 2*F*_c_^2^)/3
5251 reflections	(Δ/σ)_max_ = 0.001
325 parameters	Δρ_max_ = 1.10 e Å^−^^3^
24 restraints	Δρ_min_ = −1.19 e Å^−^^3^

### Special details

**Table:**

Geometry. All e.s.d.'s (except the e.s.d. in the dihedral angle between two l.s. planes) are estimated using the full covariance matrix. The cell e.s.d.'s are taken into account individually in the estimation of e.s.d.'s in distances, angles and torsion angles; correlations between e.s.d.'s in cell parameters are only used when they are defined by crystal symmetry. An approximate (isotropic) treatment of cell e.s.d.'s is used for estimating e.s.d.'s involving l.s. planes.
Refinement. Refinement of *F*^2^ against ALL reflections. The weighted *R*-factor *wR* and goodness of fit *S* are based on *F*^2^, conventional *R*-factors *R* are based on *F*, with *F* set to zero for negative *F*^2^. The threshold expression of *F*^2^ > σ(*F*^2^) is used only for calculating *R*-factors(gt) *etc*. and is not relevant to the choice of reflections for refinement. *R*-factors based on *F*^2^ are statistically about twice as large as those based on *F*, and *R*- factors based on ALL data will be even larger.

### Fractional atomic coordinates and isotropic or equivalent isotropic displacement parameters (Å^2^)

**Table:**

	*x*	*y*	*z*	*U*_iso_*/*U*_eq_	
I1A	0.38817 (5)	0.22093 (4)	0.17667 (3)	0.01707 (12)	
I2A	−0.17168 (6)	0.58657 (5)	0.16991 (3)	0.02695 (14)	
I3A	−0.09149 (5)	0.19121 (4)	0.46691 (2)	0.01540 (11)	
I1B	0.64018 (5)	0.26656 (5)	0.82446 (3)	0.02072 (12)	
I2B	0.38563 (5)	0.31590 (4)	0.53036 (2)	0.01505 (11)	
I3B	0.08377 (6)	−0.09395 (5)	0.82042 (3)	0.02305 (13)	
O1A	0.3577 (5)	0.1765 (4)	0.3694 (2)	0.0153 (11)	
O2A	0.1825 (5)	0.0114 (4)	0.3694 (3)	0.0165 (11)	
O3A	0.2236 (5)	0.5535 (4)	0.0837 (3)	0.0192 (11)	
O4A	0.1008 (5)	0.3729 (4)	0.0665 (3)	0.0168 (11)	
O1B	0.2646 (5)	0.1247 (4)	0.9244 (2)	0.0158 (11)	
O2B	0.3971 (5)	−0.0629 (4)	0.9177 (2)	0.0168 (11)	
O3B	0.7581 (5)	0.3230 (4)	0.6314 (3)	0.0155 (11)	
O4B	0.5834 (5)	0.4882 (4)	0.6325 (3)	0.0155 (11)	
N1A	−0.2391 (6)	0.4225 (5)	0.3477 (3)	0.0200 (14)	
H1A	−0.2936	0.4861	0.3225	0.024*	
H1B	−0.2724	0.3864	0.3968	0.024*	
N1B	0.1464 (6)	0.0829 (5)	0.6453 (3)	0.0198 (14)	
H1C	0.1508	0.1200	0.5961	0.024*	
H1D	0.0726	0.0197	0.6698	0.024*	
C1A	0.1312 (7)	0.2407 (6)	0.3123 (4)	0.0084 (14)	
C2A	0.1852 (8)	0.2955 (6)	0.2348 (4)	0.0126 (15)	
C3A	0.0967 (8)	0.3922 (6)	0.1933 (4)	0.0130 (15)	
C4A	−0.0451 (8)	0.4349 (6)	0.2320 (4)	0.0134 (15)	
C5A	−0.1032 (8)	0.3800 (6)	0.3106 (4)	0.0140 (15)	
C6A	−0.0100 (7)	0.2800 (6)	0.3494 (4)	0.0097 (14)	
C7A	0.2236 (8)	0.1295 (7)	0.3534 (4)	0.0150 (15)	
C8A	0.4678 (8)	0.0755 (7)	0.3980 (4)	0.0258 (19)	
H8A	0.5618	0.1190	0.4079	0.039*	
H8B	0.5026	0.0254	0.3599	0.039*	
H8C	0.4132	0.0141	0.4457	0.039*	
C9A	0.1392 (8)	0.4374 (6)	0.1075 (4)	0.0138 (15)	
C10A	0.2670 (9)	0.6031 (7)	0.0006 (4)	0.0276 (19)	
H10A	0.3284	0.6885	−0.0122	0.041*	
H10B	0.1689	0.6162	−0.0218	0.041*	
H10C	0.3327	0.5380	−0.0200	0.041*	
C1B	0.3607 (8)	0.1014 (6)	0.8020 (4)	0.0139 (15)	
C2B	0.4819 (7)	0.1978 (6)	0.7639 (4)	0.0129 (15)	
C3B	0.4876 (8)	0.2602 (6)	0.6861 (4)	0.0140 (15)	
C4B	0.3739 (7)	0.2233 (6)	0.6478 (4)	0.0128 (15)	
C5B	0.2547 (8)	0.1237 (6)	0.6838 (4)	0.0160 (16)	
C6B	0.2527 (7)	0.0631 (6)	0.7624 (4)	0.0138 (15)	
C7B	0.3465 (7)	0.0435 (6)	0.8873 (4)	0.0124 (15)	
C8B	0.2448 (9)	0.0829 (7)	1.0074 (4)	0.0273 (19)	
H8D	0.1836	0.1490	1.0292	0.041*	
H8E	0.3509	0.0758	1.0226	0.041*	
H8F	0.1867	−0.0042	1.0264	0.041*	
C9B	0.6117 (8)	0.3721 (6)	0.6468 (4)	0.0133 (15)	
C10B	0.8891 (8)	0.4222 (7)	0.6051 (4)	0.0260 (18)	
H10D	0.9907	0.3782	0.5952	0.039*	
H10E	0.8941	0.4700	0.6444	0.039*	
H10F	0.8715	0.4859	0.5578	0.039*	

### Atomic displacement parameters (Å^2^)

**Table:**

	*U*^11^	*U*^22^	*U*^33^	*U*^12^	*U*^13^	*U*^23^
I1A	0.0142 (3)	0.0210 (3)	0.0134 (3)	0.00420 (19)	0.00138 (19)	−0.0023 (2)
I2A	0.0343 (3)	0.0252 (3)	0.0207 (3)	0.0188 (2)	−0.0082 (2)	−0.0030 (2)
I3A	0.0146 (3)	0.0179 (2)	0.0123 (2)	0.00037 (18)	0.00006 (19)	−0.00286 (19)
I1B	0.0208 (3)	0.0254 (3)	0.0153 (3)	−0.0088 (2)	−0.0064 (2)	−0.0017 (2)
I2B	0.0154 (3)	0.0171 (2)	0.0116 (2)	−0.00127 (18)	−0.00228 (19)	−0.00199 (19)
I3B	0.0247 (3)	0.0223 (3)	0.0187 (3)	−0.0151 (2)	0.0029 (2)	−0.0032 (2)
O1A	0.010 (3)	0.019 (3)	0.018 (3)	0.006 (2)	−0.009 (2)	−0.004 (2)
O2A	0.013 (3)	0.013 (3)	0.023 (3)	0.001 (2)	−0.005 (2)	−0.002 (2)
O3A	0.023 (3)	0.019 (3)	0.013 (3)	−0.006 (2)	−0.001 (2)	0.000 (2)
O4A	0.022 (3)	0.014 (2)	0.018 (3)	−0.002 (2)	−0.008 (2)	−0.005 (2)
O1B	0.016 (3)	0.020 (3)	0.012 (3)	0.008 (2)	0.000 (2)	−0.006 (2)
O2B	0.023 (3)	0.010 (3)	0.013 (3)	0.002 (2)	−0.002 (2)	0.005 (2)
O3B	0.011 (3)	0.017 (3)	0.019 (3)	−0.0020 (19)	0.004 (2)	−0.008 (2)
O4B	0.015 (3)	0.011 (3)	0.019 (3)	0.0012 (19)	−0.003 (2)	0.000 (2)
N1A	0.021 (4)	0.022 (3)	0.014 (3)	0.015 (3)	−0.003 (3)	0.000 (3)
N1B	0.020 (3)	0.024 (3)	0.015 (3)	−0.012 (3)	−0.003 (3)	−0.004 (3)
C1A	0.005 (3)	0.010 (3)	0.011 (4)	0.000 (3)	0.001 (3)	−0.003 (3)
C2A	0.016 (4)	0.010 (3)	0.014 (4)	0.001 (3)	−0.006 (3)	−0.005 (3)
C3A	0.013 (4)	0.007 (3)	0.020 (4)	0.001 (3)	−0.005 (3)	−0.006 (3)
C4A	0.013 (3)	0.011 (2)	0.016 (3)	0.004 (2)	−0.005 (2)	−0.003 (2)
C5A	0.017 (4)	0.013 (4)	0.013 (4)	0.003 (3)	−0.001 (3)	−0.006 (3)
C6A	0.011 (2)	0.007 (2)	0.009 (2)	−0.002 (2)	0.000 (2)	−0.001 (2)
C7A	0.014 (4)	0.018 (4)	0.012 (4)	0.002 (3)	0.003 (3)	−0.005 (3)
C8A	0.022 (5)	0.022 (4)	0.038 (5)	0.009 (3)	−0.017 (4)	−0.009 (4)
C9A	0.010 (3)	0.012 (2)	0.017 (3)	0.005 (2)	−0.002 (2)	0.000 (2)
C10A	0.030 (3)	0.028 (3)	0.022 (3)	−0.007 (2)	−0.005 (2)	−0.003 (2)
C1B	0.015 (4)	0.008 (3)	0.013 (4)	0.001 (3)	0.003 (3)	0.005 (3)
C2B	0.010 (4)	0.016 (4)	0.017 (4)	0.001 (3)	−0.004 (3)	−0.009 (3)
C3B	0.013 (4)	0.012 (4)	0.012 (4)	0.000 (3)	0.003 (3)	0.003 (3)
C4B	0.013 (4)	0.010 (3)	0.014 (4)	−0.001 (3)	0.000 (3)	−0.003 (3)
C5B	0.011 (4)	0.015 (4)	0.025 (4)	0.004 (3)	−0.012 (3)	−0.007 (3)
C6B	0.010 (4)	0.007 (3)	0.023 (4)	0.000 (3)	0.001 (3)	−0.004 (3)
C7B	0.011 (4)	0.016 (4)	0.009 (4)	−0.004 (3)	0.005 (3)	−0.004 (3)
C8B	0.033 (5)	0.038 (5)	0.012 (4)	0.016 (4)	−0.001 (3)	−0.011 (4)
C9B	0.016 (4)	0.015 (4)	0.008 (4)	−0.007 (3)	−0.001 (3)	−0.002 (3)
C10B	0.008 (4)	0.031 (5)	0.040 (5)	−0.002 (3)	−0.001 (3)	−0.014 (4)

### Geometric parameters (Å, °)

**Table:**

I1A—C2A	2.110 (6)	C1A—C7A	1.502 (8)
I2A—C4A	2.096 (6)	C2A—C3A	1.394 (8)
I3A—C6A	2.110 (6)	C3A—C4A	1.412 (9)
I1B—C2B	2.111 (6)	C3A—C9A	1.504 (9)
I2B—C4B	2.109 (6)	C4A—C5A	1.410 (9)
I3B—C6B	2.103 (6)	C5A—C6A	1.416 (8)
O1A—C7A	1.341 (7)	C8A—H8A	0.9800
O1A—C8A	1.453 (7)	C8A—H8B	0.9800
O2A—C7A	1.220 (7)	C8A—H8C	0.9800
O3A—C9A	1.333 (7)	C10A—H10A	0.9800
O3A—C10A	1.461 (8)	C10A—H10B	0.9800
O4A—C9A	1.209 (7)	C10A—H10C	0.9800
O1B—C7B	1.332 (7)	C1B—C6B	1.377 (9)
O1B—C8B	1.455 (7)	C1B—C2B	1.392 (8)
O2B—C7B	1.213 (7)	C1B—C7B	1.511 (9)
O3B—C9B	1.346 (7)	C2B—C3B	1.402 (9)
O3B—C10B	1.444 (7)	C3B—C4B	1.392 (8)
O4B—C9B	1.195 (7)	C3B—C9B	1.510 (8)
N1A—C5A	1.360 (8)	C4B—C5B	1.395 (9)
N1A—H1A	0.8800	C5B—C6B	1.417 (9)
N1A—H1B	0.8800	C8B—H8D	0.9800
N1B—C5B	1.377 (8)	C8B—H8E	0.9800
N1B—H1C	0.8800	C8B—H8F	0.9800
N1B—H1D	0.8800	C10B—H10D	0.9800
C1A—C2A	1.384 (8)	C10B—H10E	0.9800
C1A—C6A	1.381 (8)	C10B—H10F	0.9800
			
C7A—O1A—C8A	115.3 (5)	O3A—C10A—H10B	109.5
C9A—O3A—C10A	114.4 (5)	H10A—C10A—H10B	109.5
C7B—O1B—C8B	115.8 (5)	O3A—C10A—H10C	109.5
C9B—O3B—C10B	114.8 (5)	H10A—C10A—H10C	109.5
C5A—N1A—H1A	120.0	H10B—C10A—H10C	109.5
C5A—N1A—H1B	120.0	C6B—C1B—C2B	120.1 (6)
H1A—N1A—H1B	120.0	C6B—C1B—C7B	120.9 (6)
C5B—N1B—H1C	120.0	C2B—C1B—C7B	119.0 (6)
C5B—N1B—H1D	120.0	C3B—C2B—C1B	119.3 (6)
H1C—N1B—H1D	120.0	C3B—C2B—I1B	119.5 (5)
C2A—C1A—C6A	121.1 (6)	C1B—C2B—I1B	120.8 (5)
C2A—C1A—C7A	118.0 (5)	C2B—C3B—C4B	119.6 (6)
C6A—C1A—C7A	120.8 (6)	C2B—C3B—C9B	119.0 (6)
C1A—C2A—C3A	119.8 (6)	C4B—C3B—C9B	121.3 (6)
C1A—C2A—I1A	120.6 (4)	C3B—C4B—C5B	122.3 (6)
C3A—C2A—I1A	119.3 (5)	C3B—C4B—I2B	119.3 (5)
C2A—C3A—C4A	118.9 (6)	C5B—C4B—I2B	118.4 (5)
C2A—C3A—C9A	120.5 (6)	N1B—C5B—C4B	122.4 (6)
C4A—C3A—C9A	120.2 (5)	N1B—C5B—C6B	121.1 (6)
C3A—C4A—C5A	122.3 (5)	C4B—C5B—C6B	116.5 (6)
C3A—C4A—I2A	118.5 (5)	C1B—C6B—C5B	122.1 (6)
C5A—C4A—I2A	119.2 (5)	C1B—C6B—I3B	118.6 (5)
N1A—C5A—C4A	122.0 (6)	C5B—C6B—I3B	119.2 (5)
N1A—C5A—C6A	121.8 (6)	O2B—C7B—O1B	124.9 (6)
C4A—C5A—C6A	116.2 (6)	O2B—C7B—C1B	125.3 (6)
C1A—C6A—C5A	121.6 (6)	O1B—C7B—C1B	109.7 (5)
C1A—C6A—I3A	120.1 (4)	O1B—C8B—H8D	109.5
C5A—C6A—I3A	118.3 (4)	O1B—C8B—H8E	109.5
O2A—C7A—O1A	124.4 (6)	H8D—C8B—H8E	109.5
O2A—C7A—C1A	124.1 (6)	O1B—C8B—H8F	109.5
O1A—C7A—C1A	111.4 (5)	H8D—C8B—H8F	109.5
O1A—C8A—H8A	109.5	H8E—C8B—H8F	109.5
O1A—C8A—H8B	109.5	O4B—C9B—O3B	125.0 (6)
H8A—C8A—H8B	109.5	O4B—C9B—C3B	124.3 (6)
O1A—C8A—H8C	109.5	O3B—C9B—C3B	110.7 (5)
H8A—C8A—H8C	109.5	O3B—C10B—H10D	109.5
H8B—C8A—H8C	109.5	O3B—C10B—H10E	109.5
O4A—C9A—O3A	125.5 (6)	H10D—C10B—H10E	109.5
O4A—C9A—C3A	122.5 (6)	O3B—C10B—H10F	109.5
O3A—C9A—C3A	112.0 (6)	H10D—C10B—H10F	109.5
O3A—C10A—H10A	109.5	H10E—C10B—H10F	109.5
			
C6A—C1A—C2A—C3A	0.2 (9)	C6B—C1B—C2B—C3B	3.2 (10)
C7A—C1A—C2A—C3A	175.5 (6)	C7B—C1B—C2B—C3B	−174.7 (6)
C6A—C1A—C2A—I1A	−173.9 (5)	C6B—C1B—C2B—I1B	176.3 (5)
C7A—C1A—C2A—I1A	1.4 (8)	C7B—C1B—C2B—I1B	−1.6 (8)
C1A—C2A—C3A—C4A	1.6 (9)	C1B—C2B—C3B—C4B	−1.0 (10)
I1A—C2A—C3A—C4A	175.7 (5)	I1B—C2B—C3B—C4B	−174.2 (5)
C1A—C2A—C3A—C9A	−170.6 (6)	C1B—C2B—C3B—C9B	175.4 (6)
I1A—C2A—C3A—C9A	3.6 (8)	I1B—C2B—C3B—C9B	2.3 (8)
C2A—C3A—C4A—C5A	−2.0 (10)	C2B—C3B—C4B—C5B	−1.2 (10)
C9A—C3A—C4A—C5A	170.1 (6)	C9B—C3B—C4B—C5B	−177.6 (6)
C2A—C3A—C4A—I2A	177.3 (4)	C2B—C3B—C4B—I2B	−179.2 (5)
C9A—C3A—C4A—I2A	−10.5 (8)	C9B—C3B—C4B—I2B	4.5 (9)
C3A—C4A—C5A—N1A	179.3 (6)	C3B—C4B—C5B—N1B	−177.1 (6)
I2A—C4A—C5A—N1A	0.0 (9)	I2B—C4B—C5B—N1B	0.8 (9)
C3A—C4A—C5A—C6A	0.7 (9)	C3B—C4B—C5B—C6B	1.3 (10)
I2A—C4A—C5A—C6A	−178.7 (4)	I2B—C4B—C5B—C6B	179.2 (4)
C2A—C1A—C6A—C5A	−1.6 (10)	C2B—C1B—C6B—C5B	−3.3 (10)
C7A—C1A—C6A—C5A	−176.7 (6)	C7B—C1B—C6B—C5B	174.6 (6)
C2A—C1A—C6A—I3A	178.6 (5)	C2B—C1B—C6B—I3B	175.1 (5)
C7A—C1A—C6A—I3A	3.5 (8)	C7B—C1B—C6B—I3B	−7.1 (8)
N1A—C5A—C6A—C1A	−177.5 (6)	N1B—C5B—C6B—C1B	179.4 (6)
C4A—C5A—C6A—C1A	1.1 (9)	C4B—C5B—C6B—C1B	1.0 (10)
N1A—C5A—C6A—I3A	2.3 (8)	N1B—C5B—C6B—I3B	1.1 (9)
C4A—C5A—C6A—I3A	−179.1 (5)	C4B—C5B—C6B—I3B	−177.3 (5)
C8A—O1A—C7A—O2A	8.7 (9)	C8B—O1B—C7B—O2B	3.7 (9)
C8A—O1A—C7A—C1A	−170.6 (5)	C8B—O1B—C7B—C1B	−178.6 (5)
C2A—C1A—C7A—O2A	−100.9 (8)	C6B—C1B—C7B—O2B	82.7 (9)
C6A—C1A—C7A—O2A	74.4 (9)	C2B—C1B—C7B—O2B	−99.4 (8)
C2A—C1A—C7A—O1A	78.4 (7)	C6B—C1B—C7B—O1B	−95.0 (7)
C6A—C1A—C7A—O1A	−106.3 (7)	C2B—C1B—C7B—O1B	82.9 (7)
C10A—O3A—C9A—O4A	1.3 (9)	C10B—O3B—C9B—O4B	8.4 (9)
C10A—O3A—C9A—C3A	−179.4 (5)	C10B—O3B—C9B—C3B	−170.5 (5)
C2A—C3A—C9A—O4A	79.0 (8)	C2B—C3B—C9B—O4B	−102.1 (8)
C4A—C3A—C9A—O4A	−93.0 (8)	C4B—C3B—C9B—O4B	74.3 (9)
C2A—C3A—C9A—O3A	−100.4 (7)	C2B—C3B—C9B—O3B	76.8 (8)
C4A—C3A—C9A—O3A	87.6 (7)	C4B—C3B—C9B—O3B	−106.8 (7)

### Hydrogen-bond geometry (Å, °)

**Table:**

*D*—H···*A*	*D*—H	H···*A*	*D*···*A*	*D*—H···*A*
N1A—H1A···I2A	0.88	2.74	3.224 (5)	116
N1A—H1A···O4B^i^	0.88	2.48	3.036 (7)	122
N1A—H1B···I3A	0.88	2.72	3.211 (5)	117
N1B—H1C···I2B	0.88	2.73	3.212 (5)	116
N1B—H1D···I3B	0.88	2.73	3.222 (5)	116
N1B—H1D···O2A^ii^	0.88	2.43	3.026 (7)	125
C8B—H8E···O2B^iii^	0.98	2.54	3.516 (9)	171
C10A—H10A···O2B^iv^	0.98	2.58	3.499 (9)	155
C10A—H10B···O4A^v^	0.98	2.54	3.519 (9)	173

Symmetry codes: (i) −*x*, −*y*+1, −*z*+1; (ii) −*x*, −*y*, −*z*+1; (iii) −*x*+1, −*y*, −*z*+2; (iv) *x*, *y*+1, *z*−1; (v) −*x*, −*y*+1, −*z*.
